# Editorial: Nutrition at the Crossroads: Food at the Intersection of Health, Environmental, Economic, and Social Sustainability—Volume II

**DOI:** 10.3389/fnut.2022.856720

**Published:** 2022-04-13

**Authors:** Kurt A. Rosentrater, Laetitia Palmade, Elif Kongar

**Affiliations:** ^1^Iowa State University, Ames, IA, United States; ^2^Polytech Montpellier, Montpellier, France; ^3^Fairfield University, Fairfield, CT, United States

**Keywords:** food, sustainability, systems, health, nutrition

With the exponential growth of the world's population, increasing awareness that our natural resources are limited, a growing food justice movement; and the development of sustainable approaches to grow, process, and distribute food is essential. The processing of raw commodities into food products and their subsequent distribution and sale, is a multi-billion-dollar industry that contributes significantly to most economies throughout the world. Meanwhile, health care costs for diet-related diseases contributes detrimentally to economies. In fact, in every country, the food industry is the largest industrial sector and consequently consumes tremendous quantities of energy, water, and other natural resources, and thus has far-reaching impacts on the environment. These impacts include greenhouse gas emissions, toxic emissions, land use changes, as well as emission of other gaseous, solid, and liquid wastes.

Historically, the criteria for food choice amongst consumers is often based on availability, taste, price, quality and safety, marketing and promotion, cultural preference, and nutritional attributes; additionally, food choices are often driven by agricultural practices and policies. However, evidence suggests that consumers are becoming increasingly aware of the environmental impacts of the foods they consume. While agricultural practices and packaging have important environmental impacts for many food products, the processing of foods, distribution, as well as food waste and loss all contribute to the environmental footprint of this industry.

This Research Topic encompasses manuscripts that focus on the interrelationships among four pillars of sustainable food supply chains (e.g., production, processing, distribution, and waste), nutrition and health (consumption), the environment (sustainability), as well as public policy. Environmental burdens and costs will also be discussed, as they cross all sectors of the supply chain, as will methodologies and techniques to calculate, model, and simulate these impacts. Thus, we will address all aspects of the Triple Bottom Line (Environmental, Social, and Economic Sustainability). Case studies of specific industry segments will also be considered, as will new technologies for improving efficiencies and decreasing environmental impacts. Government policies will also play a critical role in adoption and improvement of the food industry.

While this Research Topic series aims to span the entire food industry, this new contribution (Volume II) has a special emphasis on the intersection amongst nutrition, dietetics, and food choice vis-à-vis potential environmental, social, and health impacts. In this second volume of our Research Topic series, we have attempted to build on our first issue (see Nutrition at the Crossroads: Food at the Intersection of Environmental, Economic, and Social Sustainability) and to cover additional facets of food systems vis-à-vis sustainability.

For example, Ringling and Marquart discuss the role that land grant universities can play in promulgating sustainable food systems, especially in terms of influencing upstream and downstream actors within supply chains. Spiker et al. continues this theme, and covers online learning opportunities for dietetics education, and how sustainability can be infused into food system curricula. Marcone et al. provide an overview of factors that influence eating behaviors in Canada, specifically environmental and sociological factors, and how these can be tied to policy development. Coffigniez et al. moves the discussion downstream, and discusses perspectives on how packaging influences sustainability, and in particular, how computer modeling can be used to assess not only environmental impacts, but also food waste and loss, and their interactions with packaging materials. This Research Topic diverges from our previous one, which was focused primarily on environmental impacts. Specifically, three of the four articles in this Research Topic focus on various social aspects, and thus serve to complement our previous issue.

Indeed, improving the sustainability of food systems, and all of the various components of therein, is not easy or straightforward. Many studies tend to focus on various environmental impacts (e.g., reducing energy use, reducing carbon emissions, reducing waste, reducing plastic use, etc.) Many industries are finding success in these by installing new, energy-efficient equipment and lighting, find new uses for waste products (e.g., recycling within the plant), and conducting energy audits. More about these can be found in Volume I of our Research Topic.

The Triple Bottom Line is a good construct for approaching sustainability, as we need to consider more aspects than the environment alone. Specifically, social impacts must be considered and improved. These might include better working conditions for workers, improved housing, education, wages, child care, and health care. Other aspects could include job satisfaction and quality of life. Some of these are not easily quantified, but are active areas of research in order to define appropriate key performance indicators.

Economic aspects cannot be ignored though. Businesses tend to not implement changes that will not be profitable, even if they will reduce environmental impacts. Not only do we need to consider the profitability to the business, but ultimately to the consumer as well. Supply chain disruptions over the past few years due to unforeseen COVID impacts have illustrated this quite clearly. Thus, the Triple Bottom Line approach to simultaneous sustainability tends to be a good lens with which to focus efforts in industry.

Additional readings can be found in other Research Topics that have been published in Frontiers in Sustainable Food Systems, including articles discussing livestock production, soil carbon, modeling agro-ecosystems, urban agriculture, food waste, and many more. These can be found at https://www.frontiersin.org/journals/sustainable-food-systems#research-topics.

We would like to thank all of the contributing authors to this Research Topic. It is encouraging to see continual innovation in terms of sustainability applications and discussions. We hope that you, the reader, also find this Research Topic interesting and useful. And hopefully this helps to move the conversation forward regarding food systems, and steps that can be taken to improve environmental and social sustainability as the industry tries to achieve the goals of an abundant, nutritious, safe, and environmentally-friendly food supply.

## Author Contributions

All authors listed have made a substantial, direct, and intellectual contribution to the work and approved it for publication.

## Conflict of Interest

The authors declare that the research was conducted in the absence of any commercial or financial relationships that could be construed as a potential conflict of interest.

## Publisher's Note

All claims expressed in this article are solely those of the authors and do not necessarily represent those of their affiliated organizations, or those of the publisher, the editors and the reviewers. Any product that may be evaluated in this article, or claim that may be made by its manufacturer, is not guaranteed or endorsed by the publisher.

